# Supplementary material for: Preparation of Aminated Sodium Lignosulfonate and Efficient Adsorption of Methyl Blue Dye
Source: Materials (Basel). 2024 Feb 24;17(5):1046. doi: 10.3390/ma17051046 (PMC10934274; doi:10.3390/ma17051046)
Supplement: Supplementary file 1 [file materials-17-01046-s001.zip › materials-2852884-supplementary.pdf]

*Supporting Information*

# Preparation of Aminated Sodium Lignosulfonate and Efficient Adsorption of Methyl Blue Dye

**Li-Zhu Huo [1], Chao-Fei Guo [1,2], Zhu-Xiang Gong [1], Hao Xu [1], Xue-Juan Yang [1,2], Yu-Xuan Wang [1,2,*] and Xi-Ping Luo [1,2,*]**

[1] College of Chemistry and Materials Engineering, Zhejiang A&F University, Hangzhou 311300, China; hlz@stu.zafu.edu.cn (L.-Z.H.); chaoguo@zafu.edu.cn (C.-F.G.); 2022005@stu.zafu.edu.cn (Z.-X.G.); jasonxu@stu.zafu.edu.cn (H.X.); yangxj@zafu.edu.cn (X.-J.Y.)

[2] Zhejiang Provincial Key Laboratory of Chemical Utilization of Forestry Biomass, Hangzhou 311300, China

* Correspondence: 20190050@zafu.edu.cn (Y.-X.W.); luoxiping@zafu.edu.cn (X.-P.L.)

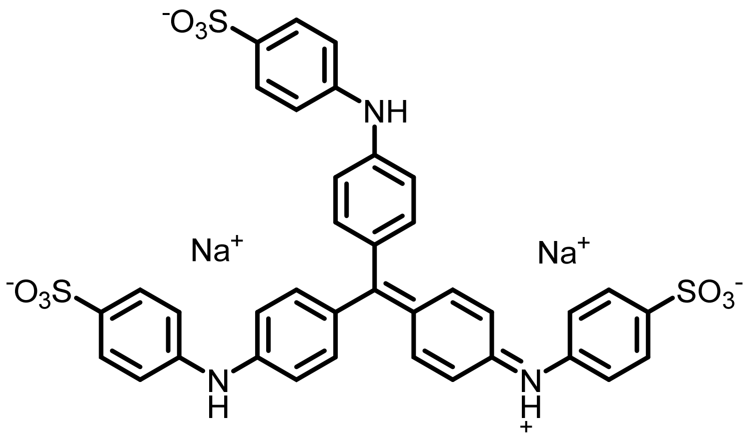


**Figure S1.** The chemical formula of methyl blue.

**Table S1**. Factors and levels of the orthogonal design.

| Levels | Factors | | | |
|---|---|---|---|---|
| | **A Temperature (°C)** | **B Time (h)** | **C DETA quantity (g)** | **D HCHO quantity (mL)** |
| 1 | 55 | 2 | 3 | 1.5 |
| 2 | 65 | 3 | 6 | 2.5 |
| 3 | 75 | 4 | 9 | 3.5 |
| 4 | 85 | 5 | 12 | 4.5 |

**Table S2**. Orthogonal experimental arrangements and removal rate.

| **Experimental NO.** | **A Temperature (°C)** | **B Time (h)** | **C DETA quantity (g)** | **D HCHO quantity (mL)** | **Removal rate %** |
|---|---|---|---|---|---|
| 1 | 55 | 2 | 3 | 1.5 | 40 |
| 2 | 55 | 3 | 6 | 2.5 | 52 |
| 3 | 55 | 4 | 9 | 3.5 | 25 |
| 4 | 55 | 5 | 12 | 4.5 | 43 |
| 5 | 65 | 2 | 6 | 3.5 | 81 |
| 6 | 65 | 3 | 3 | 4.5 | 90 |
| 7 | 65 | 4 | 12 | 1.5 | 38 |
| 8 | 65 | 5 | 9 | 2.5 | 38 |
| 9 | 75 | 2 | 9 | 4.5 | 31 |
| 10 | 75 | 3 | 12 | 3.5 | 34 |
| 11 | 75 | 4 | 3 | 2.5 | 97 |
| 12 | 75 | 5 | 6 | 1.5 | 49 |
| 13 | 85 | 2 | 12 | 2.5 | 55 |
| 14 | 85 | 3 | 9 | 1.5 | 23 |
| 15 | 85 | 4 | 6 | 4.5 | 91 |
| 16 | 85 | 5 | 3 | 3.5 | 94 |
| $K_{i1}$ | 160 | 207 | 321 | 150 | |
| $K_{i2}$ | 247 | 199 | 273 | 242 | |
| $K_{i3}$ | 211 | 251 | 117 | 234 | |
| $K_{i4}$ | 263 | 224 | 170 | 255 | |
| $k_{i1}$ | 40 | 51.75 | 80.25 | 37.5 | |
| $k_{i2}$ | 61.75 | 49.75 | 68.25 | 60.5 | |
| $k_{i3}$ | 52.75 | 62.75 | 29.25 | 58.5 | |
| $k_{i4}$ | 65.75 | 56 | 42.5 | 63.75 | |
| $R_i$ | 25.75 | 13 | 51 | 26.25 | |
| Factor primary and secondary order | | | C> D > A >B | | |
| Optimal level | $A_4$ | $B_3$ | $C_1$ | $D_4$ | |
| Optimal combination | | | $A_4B_3C_1D_4$ | | |

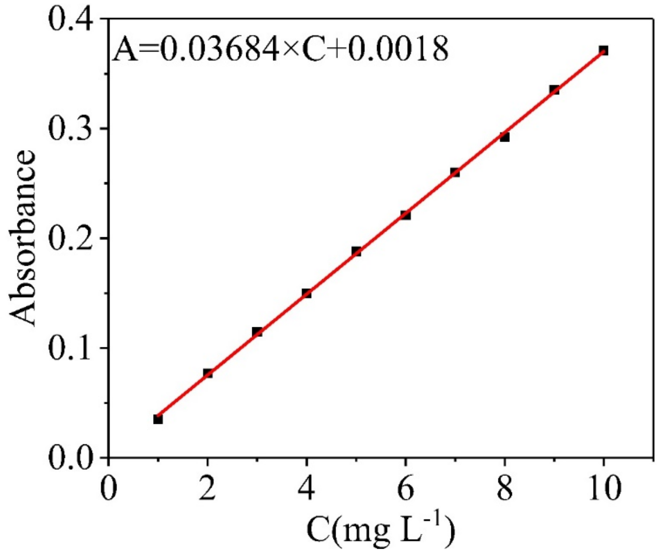


**Figure S2.** Methyl blue dye standard curve diagram.
